# Supplementary material for: Predicting central cervical lymph node metastasis in papillary thyroid carcinoma with Hashimoto’s thyroiditis: a practical nomogram based on retrospective study
Source: PeerJ. 2024 Apr 19;12:e17108. doi: 10.7717/peerj.17108 (PMC11034492; doi:10.7717/peerj.17108)
Supplement: Supplemental Information 2 [file peerj-12-17108-s002.docx]

**Codebook of categorical data**

| **Categorical data** | 0 | 1 | 2 | 3 |
| --- | --- | --- | --- | --- |
| **sex** | Female | Male |  |  |
| **age level, year** | <55 | ≥55 |  |  |
| **tumor size, mm** | ≤10 | >10 |  |  |
| **thyroid function level** |  | Normal | Hypothyroidism | Hyperthyroidism |
| **TPOAb level** |  | Low  (≤82.30 IU/ml) | High  (>82.30 IU/ml) |  |
| **TGAb level** |  | Low  (≤212.25 IU/ml) | High  (>212.25 IU/ml) |  |
| **extrathyroidal extension** | No | Yes |  |  |
| **multifocality** | No | Yes |  |  |
| **composition** | Mixed cystic and solid | Solid or almost solid |  |  |
| **echogenicity** | Hyperechoic or isoechoic | Hypoechoic or very hypoechoic |  |  |
| **margin** | Non-extrathyroidal extension | Extrathyroidal extension |  |  |
| **echogenic foci** | Non-punctate echogenic foci | Punctate echogenic foci |  |  |
| **blood** | 0 | 1 | 2 | 3 |
| **shape** | Wider-than-tall | Taller-than-wide |  |  |
| **PMTC** | No | Yes |  |  |
| **US_CLNM** | Negative | Positive |  |  |
| **US_LLNM** | Negative | Positive |  |  |
| **LNM** | Negative | Positive |  |  |
| **CLNM** | Negative | Positive |  |  |
| **LLNM** | Negative | Positive |  |  |
